# Identification and validation of a novel stress granules-related prognostic model in colorectal cancer

**DOI:** 10.3389/fgene.2023.1105368

**Published:** 2023-05-02

**Authors:** Zhihao Liu, Enen Zhao, Huali Li, Dagui Lin, Chengmei Huang, Yi Zhou, Yaxin Zhang, Xingyan Pan, Wenting Liao, Fengtian Li

**Affiliations:** State Key Laboratory of Oncology in South China, Collaborative Innovation Center for Cancer Medicine, Department of Experimental Research, Sun Yat-sen University Cancer Center, Guangzhou, China

**Keywords:** stress granules, gene signature, colorectal cancer, prognostic model, chemotherapy resistance

## Abstract

**Aims:** A growing body of evidence demonstrates that Stress granules (SGs), a non-membrane cytoplasmic compartments, are important to colorectal development and chemoresistance. However, the clinical and pathological significance of SGs in colorectal cancer (CRC) patients is unclear. The aim of this study is to propose a new prognostic model related to SGs for CRC on the basis of transcriptional expression.

**Main methods:** Differentially expressed SGs-related genes (DESGGs) were identified in CRC patients from TCGA dataset by limma R package. The univariate and Multivariate Cox regression model was used to construct a SGs-related prognostic prediction gene signature (SGPPGS). The CIBERSORT algorithm was used to assess cellular immune components between the two different risk groups. The mRNA expression levels of the predictive signature from 3 partial response (PR) and 6 stable disease (SD) or progress disease (PD) after neoadjuvant therapy CRC patients’ specimen were examined.

**Key findings:** By screening and identification, SGPPGS comprised of four genes (CPT2, NRG1, GAP43, and CDKN2A) from DESGGs is established. Furthermore, we find that the risk score of SGPPGS is an independent prognostic factor to overall survival. Notably, the abundance of immune response inhibitory components in tumor tissues is upregulated in the group with a high-risk score of SGPPGS. Importantly, the risk score of SGPPGS is associated with the chemotherapy response in metastatic colorectal cancer.

**Significance:** This study reveals the association between SGs related genes and CRC prognosis and provides a novel SGs related gene signature for CRC prognosis prediction.

## Introduction

Ranking third among all malignancies worldwide, colorectal cancer (CRC) is also the second most common cause in terms of cancer-related deaths (Keum and Giovannucci, 2019). It is crucial to diagnose CRC patients early and to use treatment strategies that improve their prognosis. Most patients, however, are diagnosed late due to the insufficiency and inefficiency of existing genetic markers and prognosis prediction models. For this reason, to ensure a non-invasive diagnosis of CRC, it is necessary to develop novel biomarkers (Deng et al., 2018; Fayazfar et al., 2019). A high level of molecular heterogeneity makes it possible for relapses and death risks to vary considerably even among patients with high similarities in clinical and pathological features (Weiser et al., 2011; Punt et al., 2017). Novel prognostic factors are therefore urgently needed to improve the accuracy of CRC patients’ risk assessments.

In response to stress stimulatory factors such as hypoxia, oxidative stress, drug administration and viral infection, stress granules (SGs) form as membrane-less organelles containing mRNA and RNA-binding proteins (Protter and Parker, 2016). The formation and dynamics of stress granules are important mechanisms that regulate the intracellular localization, translation, and degradation of mRNAs in stressful states. mRNAs that are suspended from translation under stress can be wrapped and protected from degradation (Wolozin, 2012; Jang et al., 2020; Mehto et al., 2021).

There is a reciprocal relationship between SGs formation and tumorigenesis. Several oncogenic signals, including KRAS (Grabocka and Bar-Sagi, 2016), PI3K (Heberle et al., 2019), TORC1 (Kedersha et al., 2013), and HDAC6 (Kwon et al., 2007), have been reported to promote stress granules assembly. Importantly, during the development of CRC, the rapid growth capacity induces various stresses from the tumor cells themselves or the tumor microenvironment, including hypoxia, acidic environment, and oxidative stress (El-Naggar and Sorensen, 2018). Tumor cells overcome these disadvantages through various adaptive strategies, among which SGs formation is an important way of this adaptive regulation, allowing tumor cells to survive in a hostile microenvironmental state (Song and Grabocka, 2020). It is believed that SGs are responsible for regulating tumor cell proliferation, invasion, metastasis, and drug resistance (Song and Grabocka, 2020). SGs also play an essential role in CRC progression. For instance, overexpression of Musashi1 promotes CD44^+^ CRC stem cell enrichment and stress granules formation, as well as increases the resistance of CRC cells to 5-Fluorouracil (5-Fu) (Chiou et al., 2017). In addition, KRAS mutated CRC cells are easier to form stress granules than wild-type cells, and the specific mechanism may be associated with high expression of COX2 activated by MAPK pathway (Grabocka and Bar-Sagi, 2016). However, the role of SGs-related genes in the prediction of CRC patients’ prognosis remains to be clarified.

Here we establish a prognostic multigene signature with prognostic-associated differentially expressed SGs-related genes (DESGGs) in the risk train cohort, which is further validated in the test cohorts. In CRC patients, the signature’s risk score predicts overall survival independently and is associated with inhibitory immune components abundance. Thus, the novel SGPPGS can be used as a potential biomarker to predict prognosis in CRC.

## Materials and methods

### Data acquisition

TCGA database was used to obtain RNA sequencing (RNA-seq) expression data of CRC patients as well as corresponding information regarding clinical follow-up. In total, 698 RNA-seq expression samples and clinical information on 630 patients were included in the study (including 647 CRC samples and 51 normal intestinal tissues).

### Identification of DESGGs and functional enrichment analysis

To identify differentially expressed stress granules-related genes (DESGGs), 844 genes that are related to SGs were retrieved based on GeneCards (https://www.genecards.org) by setting the threshold of the relevance score at 4 (Supplementary Table S1), and a further preprocessing step was performed using the limma package based on the TCGA samples to identify DESGGs [FDR < 0.05, log2 fold change (FC) ≥ 1]. Additionally, we excluded genes whose average count value was below 1. 223 DESGGs for further analysis were retained (Supplementary Table S2). Then, the major biological attributes of the genes were determined using gene functional enrichment analysis, such as Gene Ontology (GO) and Kyoto Encyclopedia of Genes and Genomes (KEGG) in the “ggplot2” package.

### Prognostic model construction and validation for SGPPGS

After excluding patients who have not been followed up for more than 30 days, a total of 573 patients’ expression data as well as clinical information was extracted to analyze further. Univariate cox regression was conducted to achieve a preliminary screening for DESGGs associated with prognosis. Then, the TCGA dataset of 573 CRC patients was randomly divided into two subgroups at a ratio of roughly 1:1 by using “createDataPartition” function in the “caret” R package, one was termed the risk train cohort (*n* = 288, Supplementary Table S3), and the other was termed the risk test cohort (*n* = 285, Supplementary Table S4). Prognostic models were developed using a multivariate Cox proportional risk regression analysis in the risk train cohort. For each patient, each gene’s expression values were incorporated respectively into a risk score formula that was weighted based on the regression coefficients of each gene’s expression value (Supplementary Table S5).

According to the risk score of each patient calculated above, we then classified patients into two different risk groups on the basis of their median risk scores. By using the R package “survival,” we analyzed the overall survival differences between two different risk groups. The prediction accuracy of this model was evaluated using the ROC curve.

To verify the efficacy of the SGPPGS, we used the risk test cohort as internal validation. The risk score formula was utilized to calculate patients’ risk scores. To categorize patients into two groups with different levels of risk score, the same cutoff criteria were used. To assess the prognostic value of SGPPGS, we then carried out Kaplan-Meier survival analysis and ROC curve analysis.

A clinical correlation analysis was conducted for 501 patients with CRC who had complete clinical records (Supplementary Table S6). We extracted the clinical information of patients and regression analysis was conducted with these variables and the risk score. By using the risk score and statistically significant clinical factors (age, T stage), a prognostic nomogram was developed in order to identify patients with CRC who are likely to survive 1, 3, and 5 years with the “RMS” package.

### Immune components analysis

The CIBERSORT (Newman et al., 2015; Charoentong et al., 2017) algorithm was used to assess cellular immune components between the two different risk groups. The differences in immune cell components were uncovered using a heatmap and a vioplot. Potential immune checkpoints were also retrieved from previous literature to analyze their immune inhibition.

### Clinical specimen

Primary colorectal tumor samples were obtained from Sun Yat-sen University Cancer Center Bio-bank.

### Quantitative RT-PCR

For quantitative RT-PCR (qRT-PCR), total RNA was extracted from cells using RNAiso Plus (cat# 9109, TaKaRa) and reverse-transcribed according to the manufacturer’s instructions. Based on instructions from the manufacturer, qRT-PCR reactions were conducted using CFX-384 Real-Time PCR System (Bio-Rad, CA, USA) and Universal SYBR qRT-PCR Master Mix (cat# MQ101-01, Vazyme, Nanjing, China). Calculations of relative quantitation values were made using the ΔΔCt method. qRT-PCR primers sequences were provided in Supplementary Table S6. To convert the results of qRT-PCR to risk scores, firstly, the difference of the Ct values of target genes between Ct values of GAPDH/Actin (ΔCt) were obtained. Secondly, the average of ΔCt was calculated using the ΔCt values in all samples. Thirdly, the difference of the ΔCt and average values of ΔCt (ΔΔCt) was obtained. Then the relative expression levels of target genes were calculated by indexation of 2 using the −ΔΔCt values. The relative expression levels were substitution in the risk score formula.

### Cell culture

DLD-1, HT29, and LoVo cell lines were purchased from ATCC, and cultured in RPIM-1640 medium supplemented with 10% fetal bovine serum, 100 units/mL penicillin and 100 ug/mL streptomycin. Cells treated with Sodium Arsenite (100 μM) for 1 h and subjected to Immunofluorescence assay and qRT-PCR. For drug sensitivity assay, 1 × 10^5^ cells treated with 5-Fu (100 μM) for 24 h were subjected to crystal violet staining.

### Immunofluorescence assay

1.5 × 10^5^ cells on the glass slide were washed by PBS for twice and fixed by 4% formaldehyde, then cells were treated with 0.1% triton X 100 for 10 min in room temperature after washed by PBS. Cells were incubated with G3BP1 antibody (Cat#: 13057-2-AP. Proteintech) at 4°C for 12 h after blocking with 4% Bovine Serum Albumin. After staining with primary antibody, cells were washed and incubated in dylight 488 conjugated secondary antibody. After further staining with DAPI, the cells blocked with antifade reagent (Cat#: P36930. ThermoFisher) were used to snap by laser confocal microscope (LSM980. ZEISS).

### Statistics analysis

In order to carry out statistical analyses, R software (Version 3.6.3) was utilized. To compare DESGG expression levels in cancer tissues with normal tissues, Wilcoxon statistics were used. We analyzed the association between SGs-related genes and overall survival by taking an approach of the univariate cox regression model, Multivariate Cox analyses were carried out to construct a predictive signature. Patients in two groups with different levels of risk were analyzed for survival using Kaplan-Meier and log-rank tests. The *p*-value <0.05 was considered significant.

**FIGURE 1:**
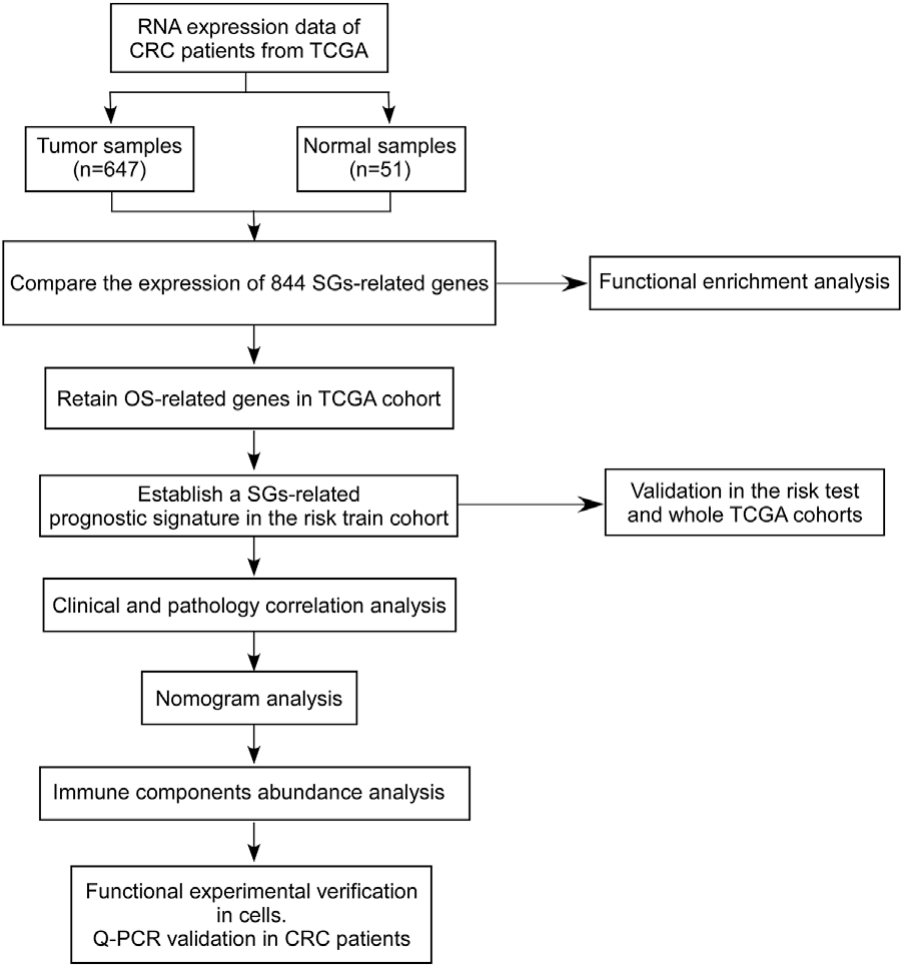
A workflow diagram of the overall analysis process.

To establish the SGs-related genes to predict prognosis in CRC, candidate SGs-related genes were obtained with differentially expressed genes analysis in the TCGA-CRC dataset (including TCGA-COAD and TCGA-READ datasets) using mRNA expression data. A training set and two test sets were used to discover and validate the ability of SGs-related signature to predict overall survival. The correlations of established SGs related predict signature between clinical pathology factors were investigated and the independency of SGs related predict signature was investigated by univariate or multivariate cox regression analyses. An overview of the analytical working flow is shown in Figure 1.

## Results

### Identification of the candidate SGs-related genes in the TCGA cohort and enrichment analysis of DESGGs

**FIGURE 2:**
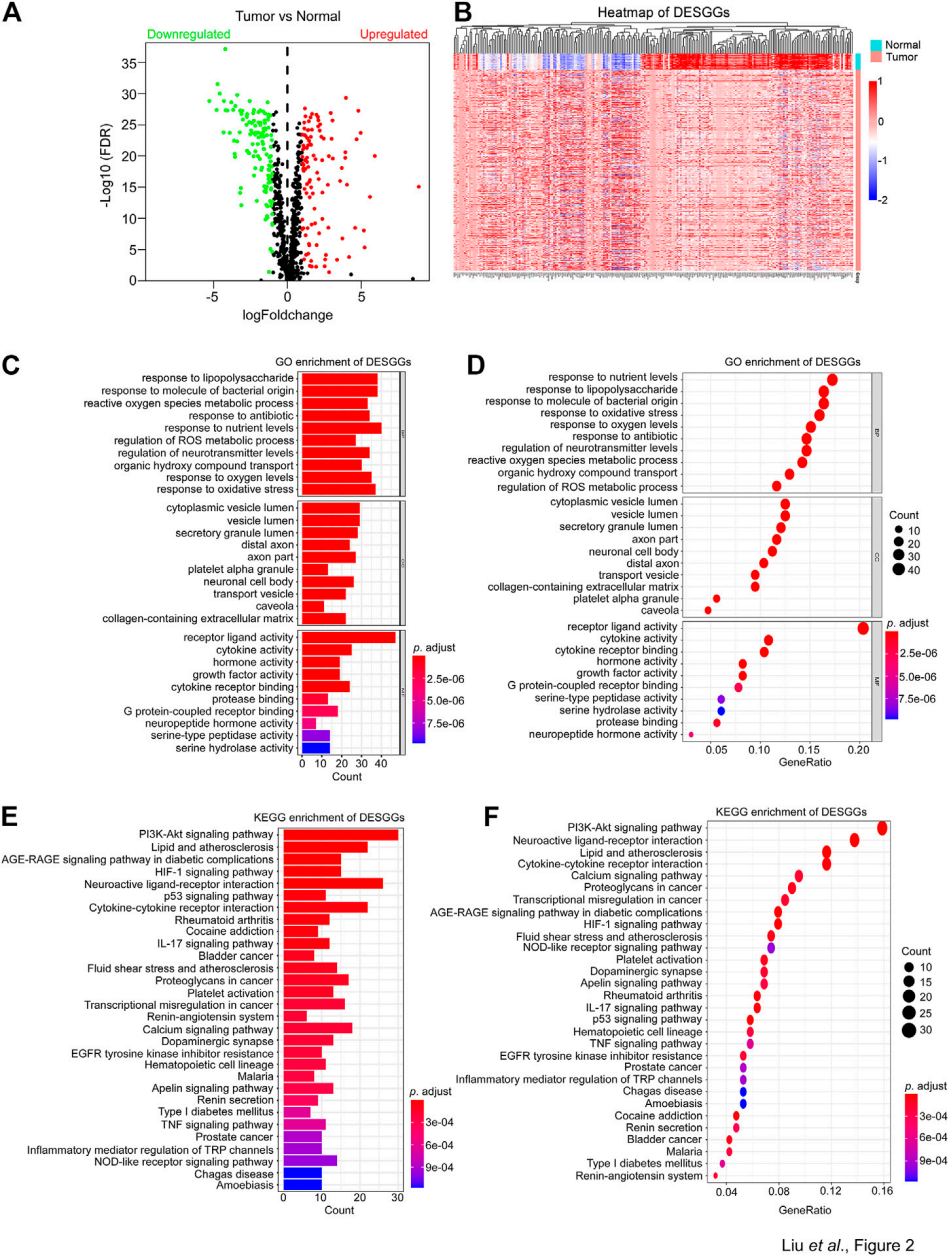
Identification of differently expressed SG genes and enrichment analysis. **(A)** 844 Stress granules-related genes were used to perform differentially expressed gene analyses between tumor and normal samples from the TCGA-CRC dataset. The 233 differentially expressed genes were identified with adjust *p*-value <0.05 and |Log2FC| > 1. A volcano plot of the DESGGs was shown. Wilcoxon statistics were used to compare the different significance between tumor and normal samples. **(B)** The mRNA levels of 233 DESGGs were used to generate a heatmap in groups of tumor and normal tissues. **(C–F)** The 233 DESGGs were used for Gene Ontology (GO) or **(E,F)** Kyoto Encyclopedia of Genes and Genomes (KEGG) enrichment analyses.

To identify the candidate genes associated with stress granules, we firstly extracted SGs-related genes from the genecard website (https://www.genecards.org/) and selected genes with a score greater than 4, for a total of 844 genes. As shown in Figure 2A, differentially expressed gene analyses showed that among 844 SGs-related genes, 127 genes were significantly downregulated in tumor tissues, whereas 106 genes were significantly upregulated. Furthermore, these 233 differentially expressed SGs-related genes (DESGGs) have different expression patterns between normal and tumor tissues in CRC (Figure 2B).

Further investigation of DESGGs’ potential function was carried out by the gene ontology analyses of the 233 DESGGs. As shown in Figures 2C, D, biological process response to an environment such as response to nutrient levels, lipopolysaccharide, oxidative stress and antibiotic were top enriched. In addition, KEGG enrichment analyses showed that the PI3K-AKT pathway, which was reported to promote SGs assembly (Heberle et al., 2019), was also top enriched (Figures 2E, F). We also performed the GSEA in TCGA CRC samples. As shown in Supplementary Figures S1A, B, oncogenic pathways including MYC, mTOR, WNT/*β*-Catenin and NOTCH were significantly enriched in tumor tissues, and pathways involved in stress response including unfolded protein response, DNA repair and UV response were also significantly enriched in tumor tissues. These results indicated that DESGGs are involved in the stress response process of tumor cells.

### Construction of SGPPGS in the risk train cohort

**FIGURE 3:**
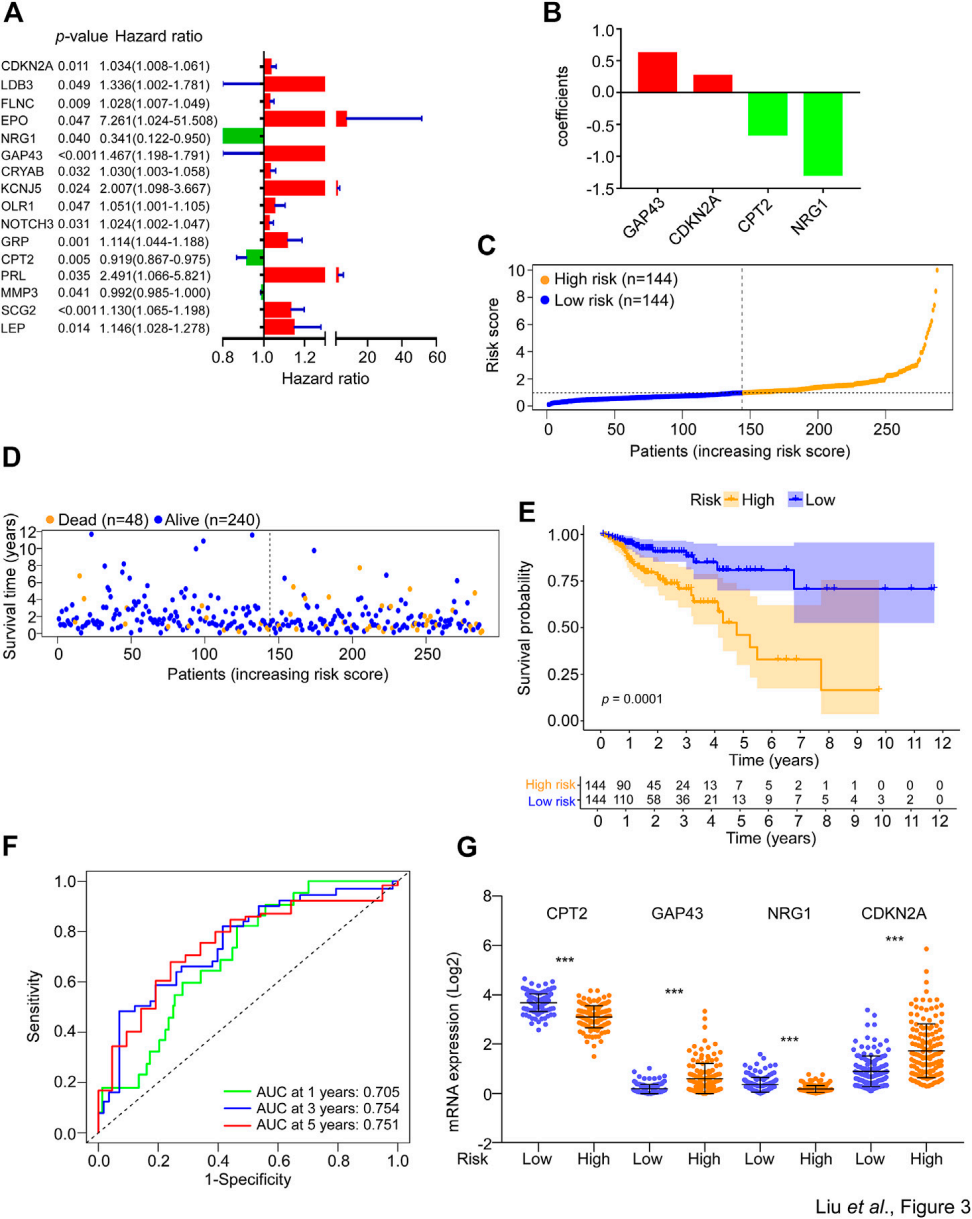
Construction of SGs-related gene signature for prognosis prediction. **(A)** The 233 DESGGs were used for univariate cox regression analyses between mRNA expression and overall survival. The hazard ratio values of the 17 genes with significant change (*p* < 0.05) were used to generate a forest plot. **(B)** The 17 genes from **(A)** were used to perform multivariate cox regression analyses between mRNA expression and overall survival. The coefficients of four optimal genes selected by the multivariate cox regression analyses were shown. **(C)** The risk score of each sample in the risk train cohort was calculated with the risk score formula. The risk score was used to generate the risk curve. **(D)** The risk score and overall survival time of each patient were used to generate a scatterplot with survival status as colored by blue (Alive, *n* = 240) or orange (Dead, *n* = 48). **(E)** Kaplan-Meier curves for the overall survival (OS) of CRC patients were stratified by risk core obtained from **(C)**. Log-rank test was used to compare the differences between the low- and high-risk groups of the risk train cohort. **(F)** The prognostic performance of the risk score was verified by AUC of time-dependent ROC curves. **(G)** The mRNA expression levels of CPT2, GAP43, NRG1, and CDKN2A in the low- or high-risk groups of the risk train cohort.

To identify prognosis-associated DESGGs, the univariate cox regression analysis was carried out by using the whole cohort of CRC patients. As shown in Figure 3A, a close correlation was found between 17 DESGGs and CRC patients’ prognosis. Furthermore, multivariate cox regression analysis among the 17 DESGGs revealed that 4 DESGGs CPT2, NRG1, GAP43, and CDKN2A comprised the predictive signature (Figure 3B). According to the coefficient value, a risk score formula was obtained as below:

Risk score = (0.620 * expression level of GAP43) + (0.262 * expression level of CDKN2A) − (0.654 * expression level of CPT2) − (1.290 * expression level of NRG1).

Thus, we defined these four genes as a SG-related prognostic predict gene signature (SGPPGS). For further investigation of the association between the SGPPGS and survival probability, the CRC patients in the risk train cohort were stratified into a high-risk group (*n* = 144) and a low-risk group (*n* = 144) according to the median cut-off value of risk score (Figure 3C). The risk score of every single patient was calculated on the basis of the risk score formula above. The expression levels of CDKN2A were upregulated whereas GAP43, CPT2 and NRG1 were downregulated in tumor tissues of CRC patients (Supplementary Figure S1C). Furthermore, the death cases were more distributed with increasing risk scores (Figure 3D). Notably, CRC patients with high risk had considerably poor survival probabilities in comparison with the low-risk patients (Figure 3E). In addition, the predictive efficacy of the risk score for overall survival was evaluated by the time-dependent ROC curve and area under the curve (AUC) of 1-, 3-, and 5-year survival were 0.705, 0.754, and 0.751, respectively (Figure 3F). The boxplot of 4 SG-related genes demonstrated that GAP43 and CDKN2A were upregulated in the high-risk group, while CPT2 and NRG1 were highly expressed in the low-risk group (Figure 3G). These results suggested that the SGPPGS was able to make a prediction on the prognosis in CRC patients.

### Validation of the SGPPGS in the risk test cohort and the whole TCGA cohort

**FIGURE 4:**
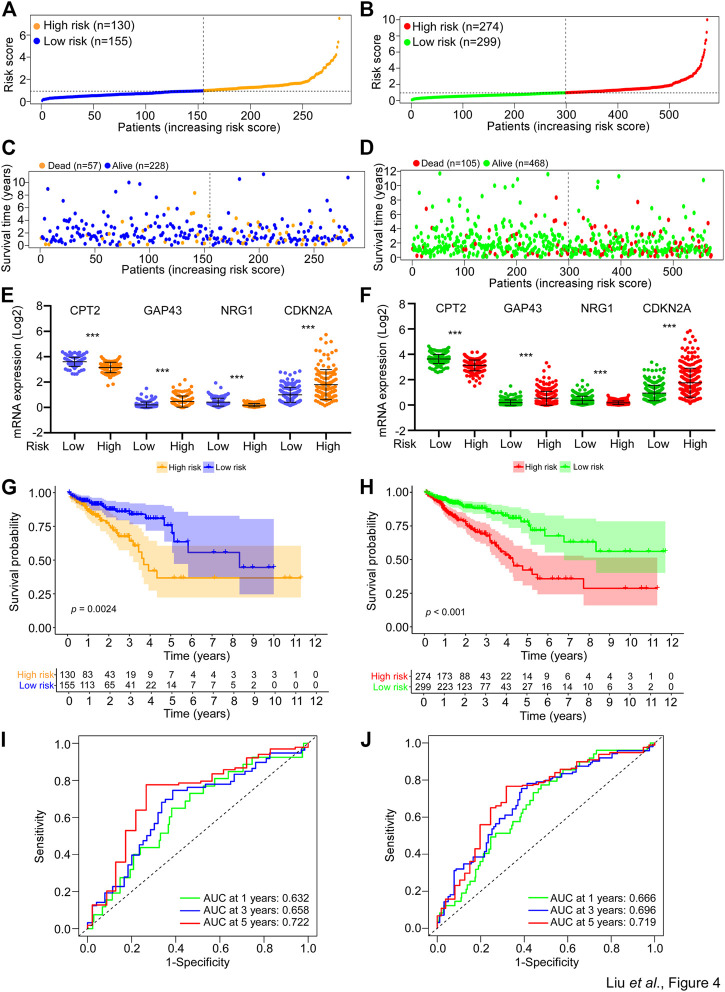
Validation of the SGPPGS in the risk test cohort and the whole TCGA cohort. **(A,B)** The risk score of each sample in the **(A)** risk test and **(B)** whole TCGA-CRC cohort was calculated based on the formula for the risk score. The risk score was used to generate the risk curve. **(C,D)** The risk score and overall survival time of each patient from the **(C)** risk test (Dead, *n* = 57; Alive, *n* = 228) and **(D)** whole TCGA-CRC cohort (Dead, *n* = 105; Alive, *n* = 468) were used to generate a scatterplot with survival status as marked by indicated color. **(E,F)** The mRNA expression levels of CPT2, GAP43, NRG1, and CDKN2A in the low- or high-risk group of risk test **(E)** and whole TCGA-CRC **(F)** cohorts. **(G,H)** Kaplan-Meier curves for the overall survival (OS) of patients from the **(G)** risk test and **(H)** whole TCGA-CRC cohort were stratified by risk core obtained from **(A,B)**. Log-rank test was used to compare the differences between the low- and high-risk groups of the two cohorts. **(I,J)** The prognostic performance of the risk score from the **(I)** risk test and the **(J)** whole TCGA-CRC cohort was verified by AUC of time-dependent ROC curve.

For the purpose of verifying the applicability of the SGPPGS for overall survival based on the risk train cohort, we performed similar analyses in the risk test cohort and the whole TCGA cohort. As shown in Figures 4A, B, the two cohorts were stratified into two groups with different levels of risk respectively by using the median cut-off value of risk score derived from the risk train cohort. Similarly, the number of death cases were elevating with increasing risk scores in the risk test cohorts (Figures 4C, D). In addition, CPT2, CDKN2A, GAP43, and NRG1 had similar expression patterns among risk test and risk train cohorts (Figures 3G, 4E, F). Notably, among these two cohorts, high-risk scores significantly predicted poor survival (Figures 4G, H). Furthermore, the predictive performance of the risk score for overall survival was evaluated by time-dependent ROC curve, and AUC of 1, 3, and 5-year survival were all higher than 0.6 (Figures 4I, J). Besides, we also used external dataset (GSE17536 from GEO database) for validation. Prognostic models using four genes (CPT2, NRG1, GAP43, and CDKN2A) showed good prognostic power in external validation sets. The area under the curve (AUC) at 1, 3, and 5 years were 0.585, 0.585, and 0.609, respectively (Supplementary Figure S1D). The prediction model could distinctly classify patients with CRC into different risk subgroups (*p* = 0.005) (Supplementary Figure S1E). These results suggested that the overall survival predictive models were reliable.

### The risk score of SGPPGS is associated with clinical features

**FIGURE 5:**
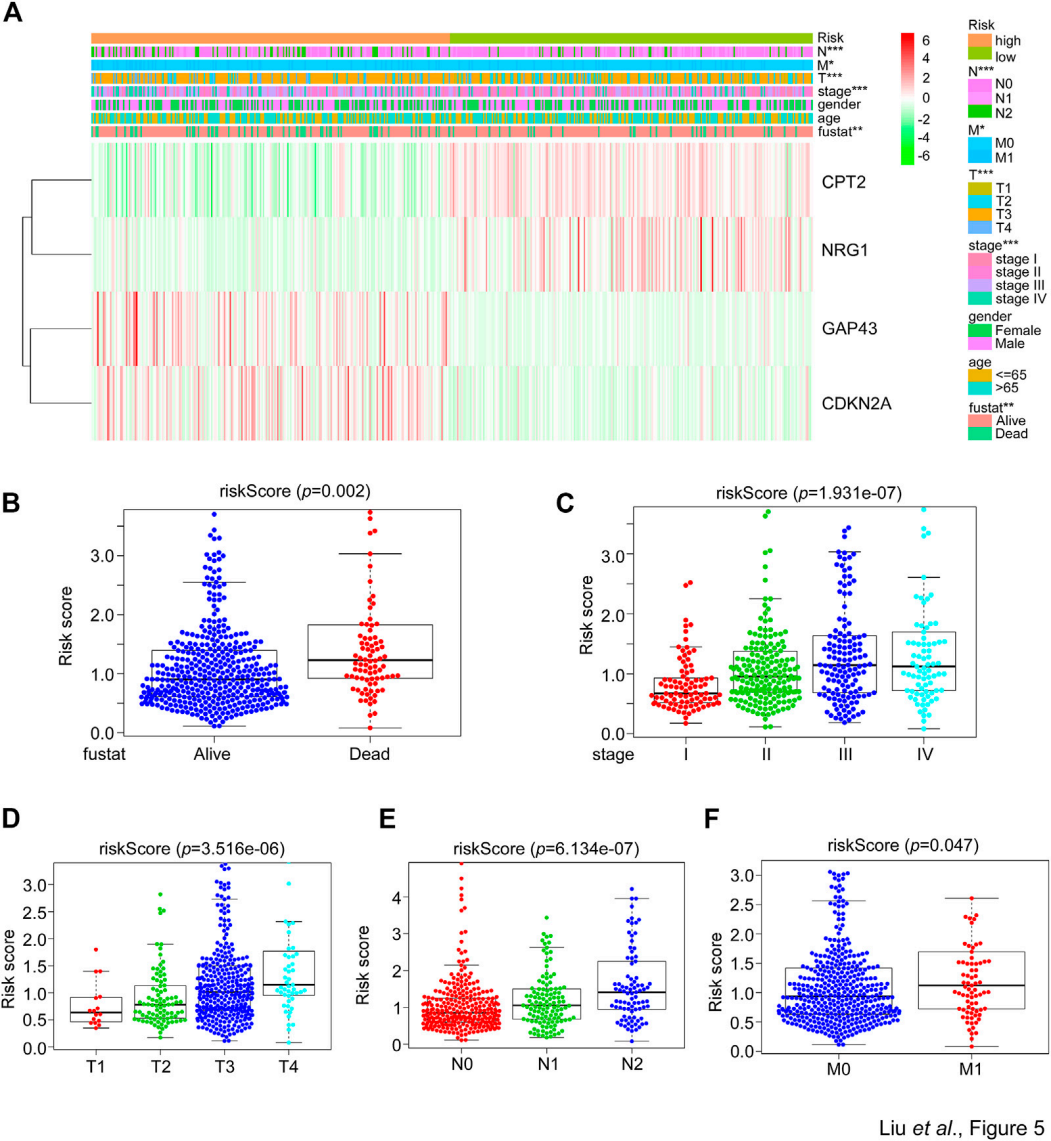
The correlation between the SGPPGS and clinical features in the TCGA cohort. **(A)** The mRNA levels of CPT2, NRG1, GAP43, and CDKN2A and clinical pathology factors between high or low-risk groups calculated by the risk score formula from the whole TCGA-CRC cohort were used to generate a heatmap in groups of tumor and normal tissues. Chi-square test was used for the comparisons. **(B–F)** The TCGA-CRC dataset was used to analyze the risk score obtained from **(A)** in **(B)** alive and dead status of patients’ tissues, and in **(C)** stage or **(D–F)** T (size and extent of main/primary tumor) N (lymph node metastasis) M (distant metastasis) related CRC tumor tissues.

The afore results suggested that the SGPPGS consisting of the four SG-related genes was reliable to make a prediction on the overall survival probability in CRC patients. But how the SGPPGS correlates with clinical characteristics remains to be investigated. As shown in Figures 5A, B, the risk score of the SGPPGS was positively associated with the death status of CRC patients. Furthermore, the risk score of the SGPPGS was also significantly associated with disease progression as characterized by pathological stage and TNM (Figures 5C–F). These results suggested that dysregulated of SGPPGS was closely associated with CRC progression.

### The SGPPGS is an independent prognostic factor

**FIGURE 6:**
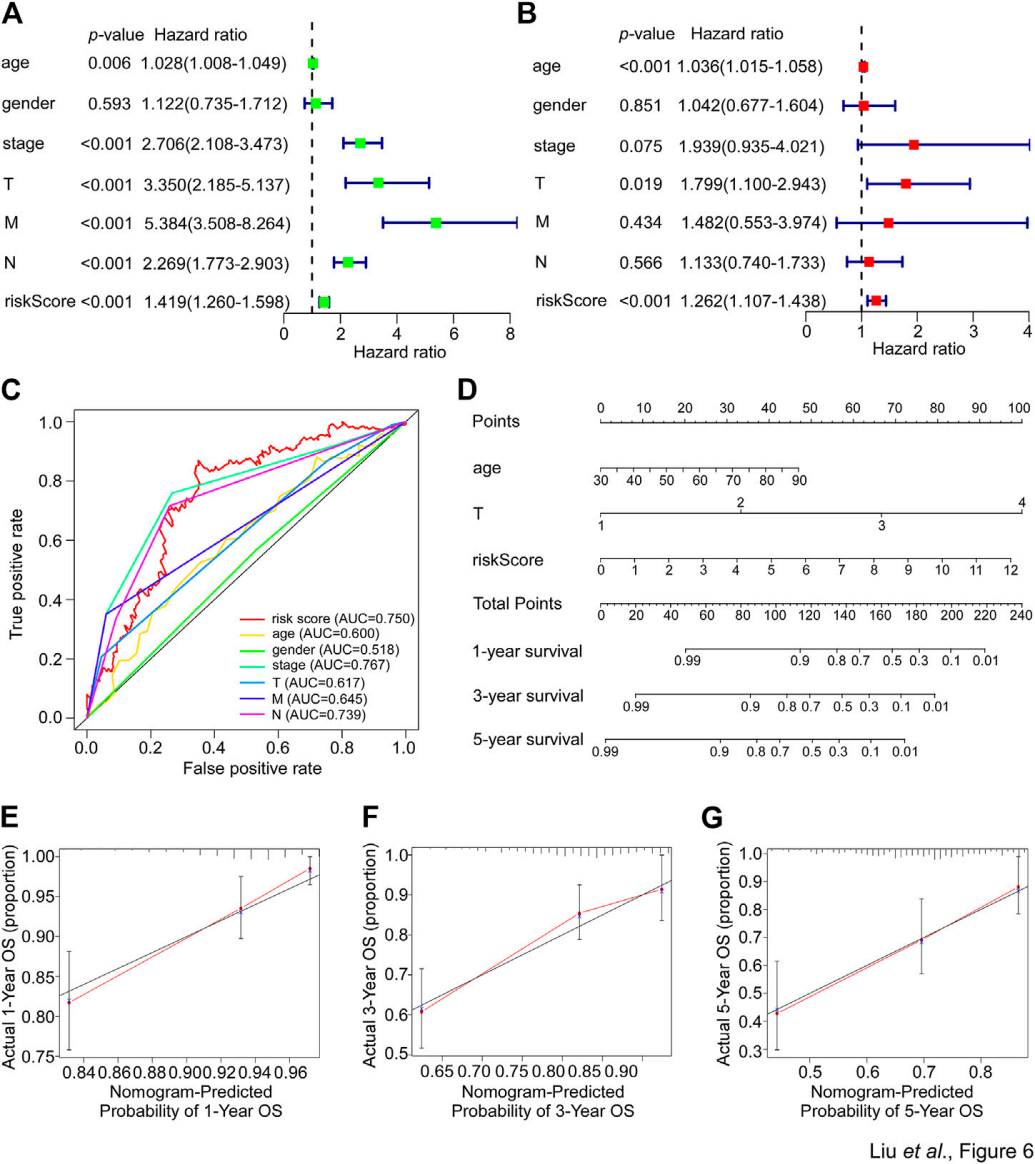
Nomogram development in the TCGA cohort. **(A)** The clinical factors and risk score calculated by the risk score formula of patients from the TCGA-CRC cohort were used for univariate cox regression analyses with overall survival. The hazard ratio values were used to generate a forest plot and the *p* values were shown. **(B)** The clinical factors and risk score calculated by the risk score formula of patients from the TCGA-CRC cohort were used for multivariate cox regression analyses with overall survival. The hazard ratio values were used to generate a forest plot and the *p* values were shown. **(C)** The prognostic performance of the clinical factors including age, gender, stage, T, N and M and risk score calculated by the risk score formula of patients from the TCGA-CRC cohort were verified by AUC of time-dependent ROC curve. **(D)** The clinical factors and risk score calculated by the risk score formula of patients from the TCGA-CRC cohort were used to perform nomogram analyses to predict 1-, 3-, 5-year overall survival of CRC patients. **(E–G)** The calibration curves test consistency between the actual overall survival rates and the predicted survival rates at 1 **(E)**, 3 **(F)**, and 5 **(G)** years.

The aforementioned data suggested a close correlation existed between the SGPPGS, clinical pathology features and poor survival probability. Thus, whether the SGPPGS is an independent indicator for CRC prognosis remains to be identified. As shown in Figures 6A, B, analyses using univariate and multivariate techniques revealed that the risk score of SGPPGS was an independent factor to predict CRC prognosis as the other clinical pathology factors including age, stage and TNM stage, whereas gender was not a prognostic factor of CRC. Furthermore, the AUC value of the risk score was 0.750, which was higher than that of other clinical factors (Figure 6C), suggesting the risk score of SGPPGS was a reliable prognostic indicator in CRC. Notably, the calibration curves showed that the constructed nomogram had a good prediction ability for 1-, 3-, and 5-year survival of CRC patients (Figures 6D–G).

### The abundance of inhibitory immune components is elevated in the group with the high risk of SGPPGS

**FIGURE 7:**
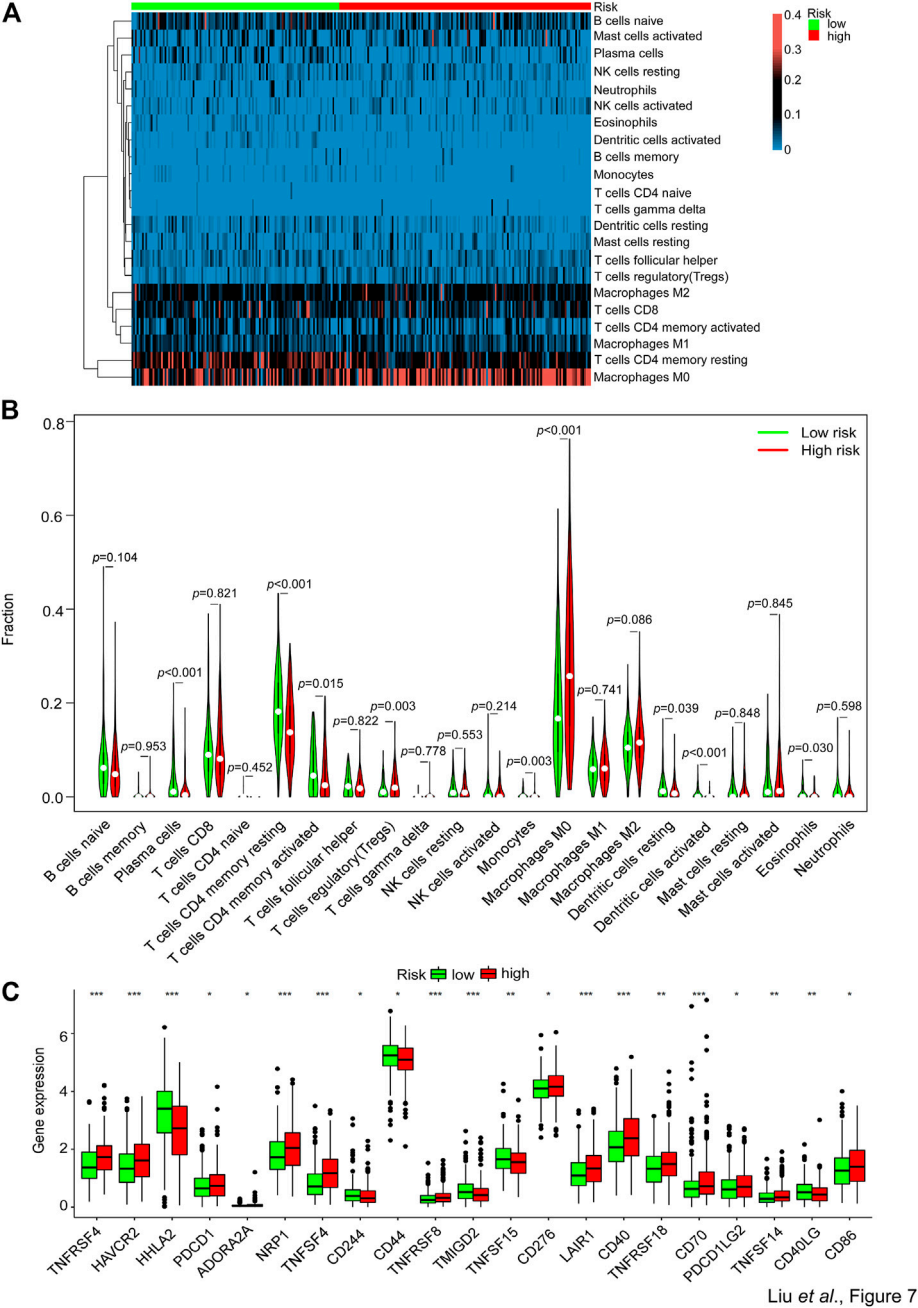
Comparison of the immune analysis between different risk groups. **(A,B)** The mRNA expression from the TCGA-CRC dataset was used to perform immune cells analyses by CIBERSORT. The immune cells’ relative fraction was used to generate the **(A)** heatmap and **(B)** vioplot in the high- or low-risk groups. Wilcox test was used to compare the relative fraction between two groups. **(C)** The whole TCGA-CRC dataset was used to analyze the mRNA expression levels of selected immune checkpoints genes in high- and low-risk groups. Adjusted *p* values were shown as: ns, not significant; *p < 0.05; **p < 0.01; ***p < 0.001.

For further exploration of the relationship between the risk score and immune components, we performed the heatmap and vioplot of immune responses based on CIBERSORT algorithms. The results indicated that the number of Treg cells, monocytes, and M0 macrophages, was seen to have a significant increase in the high-risk group versus the low-risk group. Conversely, the number of plasma cells, memory resting CD4^+^ T cells, memory activated CD4^+^ T cells, resting dendritic cells, activated dendritic cells and eosinophils was significantly lower in the high-risk group than in the low-risk group (Figures 7A, B). Notably, the expression of a series of immune checkpoint genes was upregulated in the high-risk group, including PD-1 and CD276 (B7-H3) (Figure 7C). These data indicated that patients with high risks tended to be in an immune inhibitory state.

### The risk score of SGPPGS is associated with limited response to neoadjuvant therapy in metastatic CRC

Afore results demonstrated that the risk score of SGPPGS was associated with a poor survival prognosis of CRC patients. Meanwhile, stress granules-associated pathways can be triggered by chemotherapy treatments. However, we still do not know what role the SGPPGS plays in chemotherapy response.

**FIGURE 8:**
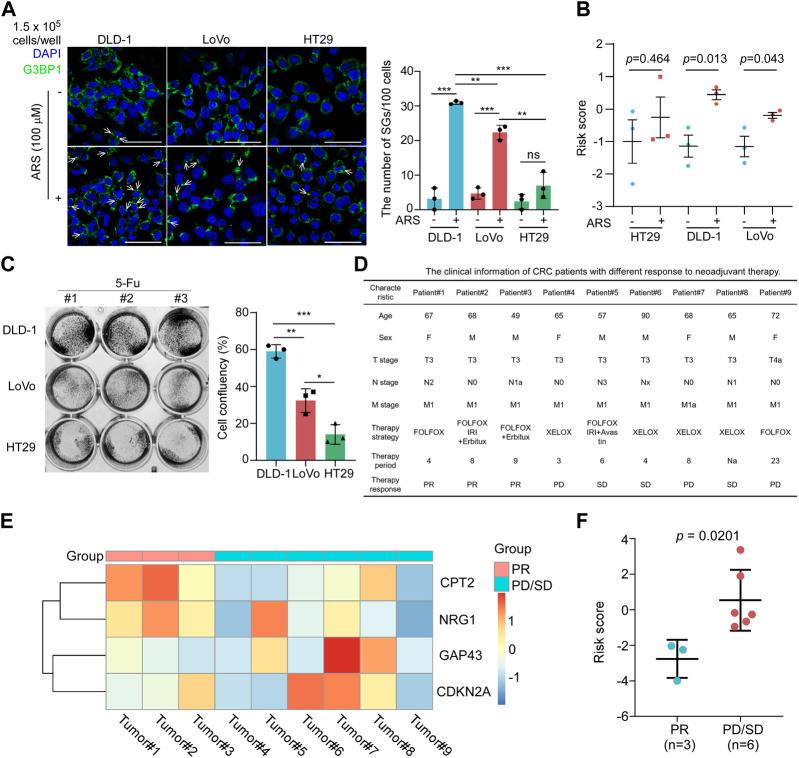
The risk score of SGPPGS is associated with chemotherapy response. **(A,B)** HT29, DLD-1, and LoVo colorectal cancer cells were treated with or without Sodium Arsenite (100 μM) for 1 h and subjected to **(A)**. Immunofluorescence assay using antibody specific for G3BP1. The white arrows indicated the SGs as characterized by assembly signaling of G3BP1 in cytoplasm, and qRT-PCR, then the mRNA expression levels of CPT2, NRG1, CDKN2A, and GAP43 were used to calculate the risk score, and **(B)** the quantitive results plot was shown (Scale bar = 50 μm). **(C)** DLD-1, LoVo, and HT29 cells were treated with 5-Fu (100 μM) for 24 h, then subjected to crystal violet staining. Unpaired two-tailed Student’s *t*-test was used for comparison between two groups. Data were presented as means ± SD. *p < 0.05; **p < 0.01; ***p < 0.001. **(D)** The clinical information of CRC patients with different response to neoadjuvant therapy. **(E,F)** The primary tumor samples from metastatic colorectal cancer patients as described in **(D)** were subjected to qRT-PCR analyses. The mRNA expression levels of CPT2, NRG1, CDKN2A, and GAP43 were used to generate the heatmap **(E)** and were used to **(F)** calculate the risk score using the risk score formula. Unpaired two-tailed Student’s *t*-test was used for comparison between two groups. Data were presented as means ± SEM.

In this way, we examined the ability of SGs formation in CRC cells. It is well established that SGs can be characterized as assembly signaling of G3BP1 in cytoplasmic puncta (Grabocka and Bar-Sagi, 2016). As shown in Figure 8A, the number of SGs as stained by anti-G3BP1 antibody by immunofluorescence assay was significantly increased in DLD-1 and LoVo cells treated with sodium arsenite (ARS), a widely used agent inducing SGs formation by inducing oxidative stress within 1 h (Grabocka and Bar-Sagi, 2016). However, there was little SGs formed in HT29 cells. Furthermore, the risk score of DLD-1 and LoVo cells was both significantly increased with ARS treatment (Figure 8B). These results suggested that the risk score of SGPPGS was associated with SGs formation in CRC cells. The first-line chemotherapy drug 5-Fu for CRC treatment is reported to induce SGs formation, which is associated with drug resistance (Kaehler et al., 2014). Notably, DLD-1 cell line was most resistant to 5-Fu treatment than LoVo and HT29, and the HT29 cell line was most sensitive to 5-Fu (Figure 8C). To further investigate the role of SGPPGS in CRC, the mRNA expression of SGPPGS in tumor samples from 9 metastatic colorectal cancer patients who accepted neoadjuvant chemotherapy was examined. As shown in Figure 8D, 3 patients (#1–#3) had a partial response (PR) and 6 patients (#4–#9) had progress disease (PD) or stable disease (SD) after neoadjuvant chemotherapy. Furthermore, the mRNA levels of CPT2 and NRG1 were upregulated in tumors from the PR group whereas CDKN2A and GAP43 were upregulated in tumors from the PD/SD group (Figure 8E). Importantly, the risk score of SGPPGS in the PD/SD group was significantly higher than PR group (Figure 8F). Similar results were also observed in TCGA CRC specimen (Supplementary Figure S1F; Supplementary Table S4). These results suggested that the prognostic model may also be applicated to predict the response rate to chemotherapy, which is consistent with the SGs’ promotion of tumor cells under chemotherapy exposure to survive in hostile environments and facilitate drug resistance.

## Discussion

Globally, there is a high prevalence of CRC among all types of cancers, with an annual global average of 1.8 million new cases diagnosed (Keum and Giovannucci, 2019). Approximately 9,00,000 people die from CRC every year since it is often diagnosed at an advanced stage (Keum and Giovannucci, 2019). Despite the therapeutic combination of targeted and cytotoxic drugs that have been applicated in CRC treatment over the past few years (Biller and Schrag, 2021), the overall survival of CRC patients is still far from satisfaction. Thus, it is important to understand the molecular mechanisms contributing to tumorigenesis and malignant progression in CRC. Chemotherapeutic response rate is associated with SGs, which can be induced by chemotherapy (Lin et al., 2019; Shi et al., 2019; Zhao et al., 2021; Wang et al., 2022). CRC cells can form SGs under strict conditions to survive (Grabocka and Bar-Sagi, 2016). In particularly, activate mutation of KRAS promotes SGs formation under oxaliplatin or ARS treatment via inactivation of eIF4A in CRC (Grabocka and Bar-Sagi, 2016). In this study, we identified the four SGs-related genes (CPT2, CDKN2A, NRG1, GAP43) that were composed of a reliable prognosis prediction gene signature and revealed the comprehensive roles of these four genes in the development of CRC. Importantly, as SGs play an important role in promoting drug resistance, a high-risk score of SGPPGS is consistently associated with limited response to neoadjuvant chemotherapy in metastatic CRC as evidenced by our transcriptional validation.

In the past decades, clinical outcome factors are widely used for prognosis prediction, such as TNM categories, tumor stage and grade (Woodard et al., 2016; Liu et al., 2021). However, novel factors for prognosis prediction are need to improve the efficacy of prediction. Stress granules, a new dimension in selective mRNA translation, have attracted considerable interest among academics since a few years ago. However, the role of stress granules in the field of cancer remains to be deciphered. In conjunction with RNA-seq and microarray techniques, multiple gene signatures-based risk scoring systems have become increasingly popular for predicting cancer prognosis (Chen et al., 2007; Zhang et al., 2013; Wang et al., 2019). It has been suggested that a number of genes might be involved in the regulation or formation of SGs in CRCs based on preliminary research (Grabocka and Bar-Sagi, 2016; Chiou et al., 2017; Legrand et al., 2020), their correlations with CRC patients’ overall survival remain largely unknown, which might be a possible explanation for the survival differences among CRC patients. As an additional benefit, prognostic models based on SGs-related genes may offer new therapeutic targets.

There is evidence that four SGs-related genes are associated with cancer. CPT2 is located on the mitochondrial membrane, where this enzyme is crucial for fatty acid oxidation (Guo et al., 2017). It was found that CRC tissue expressed decreased levels of CPT2, which was consistent with our study (Guo et al., 2017; Zhang et al., 2017). Patients with CRC may have a better prognosis if CPT2 is expressed highly in their cancer tissues (Guo et al., 2017). Mechanistically, as a result of CPT2 downregulation in CRC, proliferation is promoted and apoptosis is inhibited via the TP53 pathway (Liu et al., 2022). Moreover, Colorectal cancer stemness and oxaliplatin resistance are induced by CPT2 downregulation, which potentiates glycolytic metabolism mediated by ROS/Wnt/*β*-Catenin pathways (Li et al., 2021). As an important peptide growth factor, neuregulin 1 (NRG1) is also a member of the family of epidermal growth factor (EGF) (Finigan et al., 2011). In common with other EGF members, the original expression of NRG1 serves as a transmembrane precursor, whose extracellular region contains the mature and soluble form (Finigan et al., 2011). Some NRG1 isoforms have been identified, including NRG1α, and NRG1β. In prostate cancer, the tumor microenvironment-derived NRG1 activates the HER3 gene to promote antiandrogen resistance (Zhang et al., 2020). Additionally, NRG1 has been shown to promote the progression in breast cancer (Tsai et al., 2003; Cheng et al., 2009; Momeny et al., 2015; Shu et al., 2022). NRG1III was upregulated in CRC but its exact role in CRC is uncertain (Guo et al., 2018). GAP43 was shown to be a “growth” or “plasticity” protein, promoting neuronal growth and regenerating axons (Piontek et al., 2002; Hocquemiller et al., 2010; Zhao et al., 2012). GAP43 accelerates the malignant development of thyroid cancer cells through epithelial-mesenchymal transition (Zheng et al., 2019) and GAP43 was also reported to be associated with metastasis promotion in lung cancer (Zhang et al., 2018). Interestingly, despite the fact that CRC tumor tissues have a reduced level of GAP43 compared with adjacent tissues, overexpression of GAP43 induces expression of ABC transporters, which are responsible for drug resistance (Chen et al., 2021). Notably, overexpression of GAP43 inhibits eIF2-mediated ribosome signaling, which is responsible for stress granules assembly (Chen et al., 2021; Montero and Trujillo-Alonso, 2011). CDKN2A is reported to reduce the level of ROS in melanoma cells. Due to this phenomenon, CDKN2A putatively promotes cell fitness under oxidative conditions (Jenkins et al., 2011). Importantly, CDKN2A is previously identified to be associated with poor prognosis in CRC (Han et al., 2020; Shao et al., 2021; Kang et al., 2022).

Together, the novel prognostic model established by four SGs-related genes is an independent prognostic factor, providing a further understanding of multi SGs-related genes mediated prognosis prediction of CRC. This study also provides a comprehensive correlation analysis among SGs-related genes and overall survival in CRC.

## Conclusion

Collectively, this study identifies four SGs-related genes that are closely related with the survival of patients in CRC and provides a novel SGs related prognostic gene signature for CRC prognosis prediction and chemotherapy response, which inspire further researches on new biomarkers and personalized therapies for colorectal cancer.

## Data Availability

Publicly available datasets were analyzed in this study. This data can be found here: https://portal.gdc.cancer.gov/ COAD and READ datasets of TCGA (The Cancer Genome Atlas). GSE17536 dataset was used for external validation and can be found in GEO database (https://www.ncbi.nlm.nih.gov/gds).

## Supplementary material

SUPPLEMENTARY FIGURE S1
**(A,B)** The mRNA expression of colorectal cancer specimens from TCGA database was used for gene set enrichment analysis in tumor and normal samples. **(C)** The heatmap showed the expression of four signature genes between tissues from tumors and normal tissues across the entire TCGA cohort. **(D,E)** ROC curve and survival analysis in GSE17536 dataset from GEO database for external validation. **(F)** The risk score in colorectal cancer specimen derived from TCGA database. The clinical outcome information was provided by cBioPortal database (https://www.cbioportal.org/).

## Abbreviations

AUC, area under the curve; BP, biological process; CC, cellular components; CRC, colorectal cancer; DESGGs, differentially expressed SGs-related genes; EGF, epidermal growth factor; GO, gene ontology; KEGG, kyoto encyclopedia of genes and genomes; M, distant metastasis; MF, molecular function; N, lymph node metastasis; PR, partial response; PD, progress disease; RNA-seq, RNA sequencing; ROC curve, receiver operating characteristic curve; SD, stable disease; SGs, stress granules; SGPPGS, SGs-related prognostic prediction gene signature; T, size and extent of main/primary tumor; TCGA, the cancer genome atlas; TNM system, tumor–node–metastasis system.
