# Supplementary figures and images for: Identification and validation of a novel stress granules-related prognostic model in colorectal cancer
Source: Front Genet. 2023 May 2;14:1105368. doi: 10.3389/fgene.2023.1105368 (PMC10187888; doi:10.3389/fgene.2023.1105368)

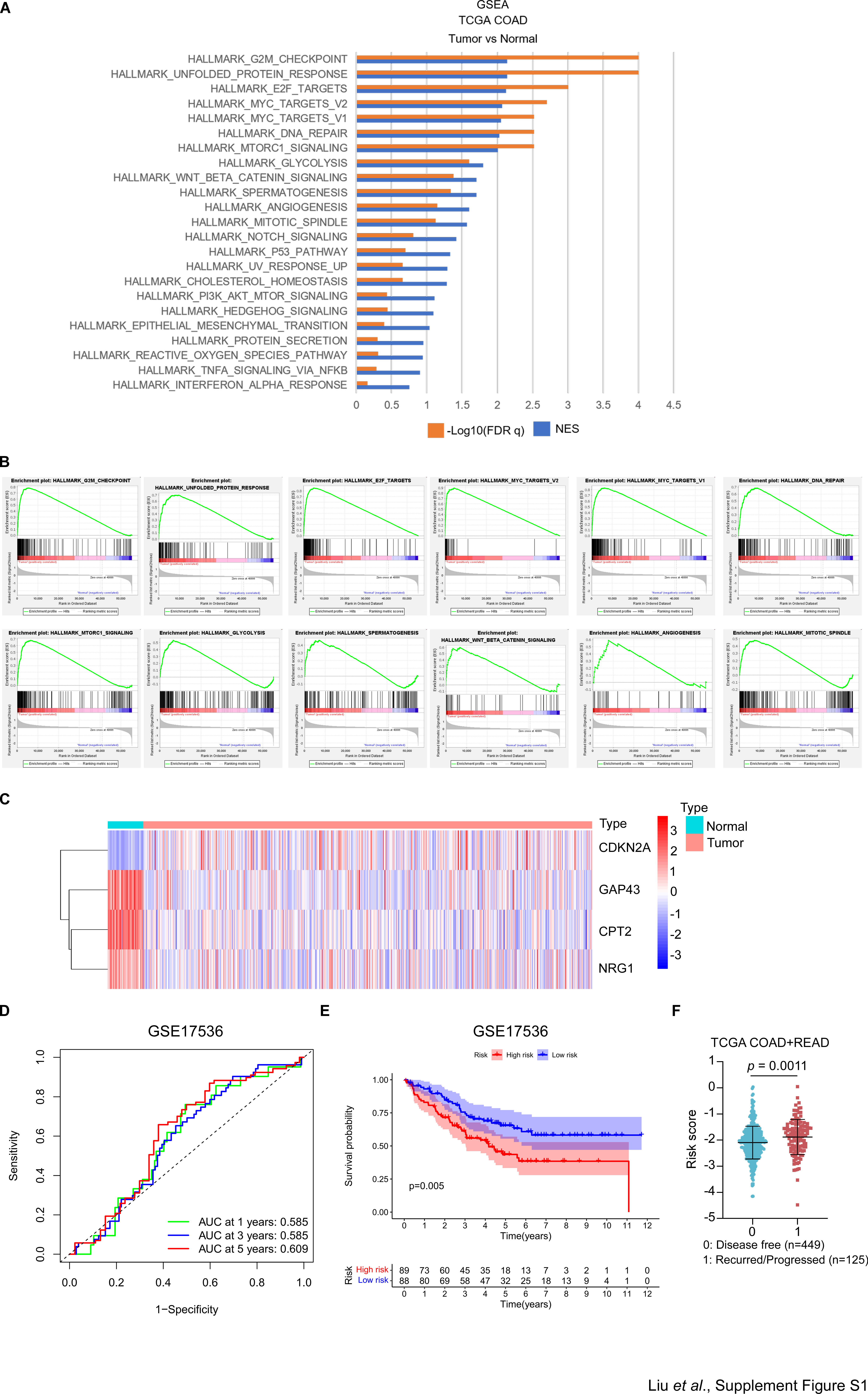

Supplement: Supplementary file 6 [file Image1.TIF]
